# Quarantine

**Published:** 1921-11

**Authors:** 


### QUARANTINE.

(By A Port Medical Officer of Health.)

The old system of isolating immigrants for forty days and forty nights, in other words, the old and valuable practice of quarantine, is no longer possible in these days when time is money. Our ports, however, are still the first line of defence against imported diseases, and the inspection of ships and their crews and of aliens and other passengers entering this country has often been valuable in preventing the importation of disease from abroad.

Separated as we are only by the narrow seas from the Continent of Europe, it is not unlikely that disease may creep past the barriers of our ports, to develop a few days later among the passengers who have scattered to the homes. If we were like the United States of America, separated from infected areas by a long sea voyage, we should not be so likely to acquire the infection carried in by aliens and others; for they would develop their diseases while they were yet on board ship in the course of the voyage, and be isolated on arrival instead of spreading disease broadcast throughout the land.

The ideal condition in port sanitary work is for every ship that arrives after a long voyage to carry perfectly healthy passengers and crew. This can be obtained to a considerable degree by quarantining and disinfecting the passengers and their goods before embarkation. The United States of America, whose standards and attainments in public health are so high, have issued certain regulations, which are of more than usual interest, dealing with the quarantine of immigrants *before* embarkation to America from European and other ports. With cholera, typhus, and smallpox endemic in Europe, these regulations must be of the greatest value, and do much to ensure that ships which arrive at American ports are free from these diseases. It is assumed that first-class passengers have not been exposed unduly to infection prior to embarkation, and that they are people of education and of clean habits. It is to deal with the rag-tag and bob-tail of Europe, who especially are prone to find their ways to the States, that the regulations are framed.

They appear to be so valuable a contribution to the practical prevention of epidemics, that we reproduce in full the sections dealing with vaccination, disinfestation, and disinfection at ports of embarkation.

I. Vaccination.

(*a*) Steerage passengers, whatever their origin, shall be vaccinated prior to embarkation unless they show satisfactory evidence of having acquired immunity to smallpox by previous attack or a successful vaccination within one year.

Medical certificates of vaccination shall not be accepted as evidence of a person's immunity to smallpox. Actual examination of every individual for marks and signs of a *successful* vaccination shall be required.

**Quarantine - (*cont.*).**

(*b*) Each steerage passenger shall receive a card, as required by paragraph 109 of the United States Quarantine Regulations. This card, stamped by the Medical or Consular Officer, shall be issued to every member of a family as well as to the head thereof.

(*c*) Members of the crew shipped in ports where smallpox prevails shall be vaccinated as in the case of steerage passengers.

(*d*) When it appears that second-class passengers who would ordinarily be third class (steerage) are travelling second class either to avoid quarantine regulations or because transportation has been provided for them, they should be vaccinated unless rendered immune by a previous attack of smallpox or a successful vaccination within one year.

II. Inspection—Delousing—Detention.

(*a*) All steerage and second-class passengers originating in countries east and south of Germany, Switzerland, and Italy, or coming from Asiatic or African Mediterranean or Black Sea ports, shall be deloused and placed under observation in clean quarters for a period of not less than twelve days prior to embarkation.

All steerage and second-class passengers who have been in such contact, prior to delousing and disinfection, with the classes of passengers subject to twelve days' detention as to render them liable to have become infested, shall also be detained twelve days under observation.

During the period of detention, passengers shall be kept in separate quarters and subjected to a daily medical inspection. While "observation" does not mean the absolute isolation of the passengers, it is intended to safeguard them against the possibility of reinfestation. Care should be taken that all passengers who are subject to detention, presenting themselves for the final examination on the day of embarkation, have actually been detained twelve days subsequent to delousing.

(*b*) Steerage passengers originating in Continental Europe, west of the easterly boundaries of Germany, Switzerland, and Italy, shall be deloused and their effects disinfected.

Second-class passengers from the same territory, and steerage and second-class passengers from Great Britain, Denmark, Norway, and Sweden, shall be inspected, and if found to have lice or eggs on their persons or clothing, shall be deloused and their effects disinfected.

(*c*) The United States Public Health Officer shall examine each individual of the steerage and second-class passengers separately on the day of embarkation; he may be assisted in this examination by one or more experienced male or female assistants.

(*d*) He shall inspect the quarters occupied by the steerage and second-class passengers aboard the vessel for the purpose of ascertaining that the bedding and quarters are free from lice or the eggs of the same, or that measures adequate for the destruction of said lice and eggs have been adopted and performed.

In so far as is practicable, such an inspection shall be made of quarters and bedding in the hotels accommodating clean passengers awaiting embarkation.

III. Disinfection.

(*a*) The baggage, both hand and hold baggage, of passengers mentioned in Article II. (*a*) shall be disinfected by steam under pressure and appropriately labelled; the baggage of Continental steerage passengers shall be disinfected in similar manner; the baggage of other steerage and second-class passengers who are found, on inspection, to be infested with vermin, shall also be disinfected.

(*b*) It should be pointed out to the steamship companies concerned that the diinfection by steam, under pressure, of bedding, blankets, and clothing from typhus- and smallpox-infected countries is as important an obligation as the delousing of the passengers and their body clothing.

In no instance should this class of baggage of second- and third-class passengers escape disinfection.

If the facilities at a port are inadequate for the purpose, the companies should take the proper steps to prohibit the bringing of such baggage; they can well do so on the ground that it is a serious menace to the health of the passengers and to the public health of the United States.

All personal effects (baggage) that have been disinfected shall be labelled to show where and when the work was done.

But it is not only the aliens who are travelling directly from Europe to the States to whom these regulations apply. Emigrants from Europe may travel by devious ways, passing, say, from Poland to Germany, and hence to Denmark or England *en route* to Canada, and enter the States from some Canadian town. The regulations deal with these wanderers, as with the more direct travellers, as follows:—

Certification of Transit Passengers.

(*a*) Steerage and second-class passengers departing for the United States via British, Danish, Norwegian, and Swedish ports may be deloused, detained, and have their effects disinfected at any of the following ports—namely, Danzig, Hamburg, Bremen, Rotterdam, and Antwerp—provided that adequate measures are adopted by the companies concerned to prevent contact between them and verminous emigrants while transferring to the ports of embarkation. Individual delousing and detention certificates should be issued to such passengers for the information and guidance of the sanitary officers in the ports and places through which they will pass.

(*b*) Steerage and second-class passengers ultimately destined for the United States via Canada shall be subjected to exactly the same measures as are required of persons sailing directly for ports of the United States.

The rigid enforcement of the provisions outlined above will certainly prevent the introduction of disease into the United States, and the authorities who framed these regulations are to be congratulated on their foresight.

